# Relationship between home garden ownership and the consumption of fruits and vegetables

**DOI:** 10.1017/S1368980024000272

**Published:** 2024-01-23

**Authors:** Mahama Saaka, Simon Awini, Fred Kizito, Eric Nang

**Affiliations:** 1 University for Development Studies, School of Allied Health Sciences, P O Box 1883, Tamale, Ghana; 2 Ghana Health Service, Wa West District Health Administration, Wechau, Ghana; 3 International Institute of Tropical Agriculture (IITA), Tamale, Ghana; 4 Ghana Health Service, Nadowli-Kaleo District Health Administration, Nadowli, Ghana

**Keywords:** Home gardens, Fruit and vegetable consumption, Knowledge scores, Northern Ghana

## Abstract

**Objective:**

This study assessed the extent to which access to home gardens associate with the frequency of fruit and vegetable (FV) consumption.

**Setting:**

The study was carried out in fifty rural communities in Northern Ghana where food insecurity and malnutrition including micronutrient deficiencies are prevalent.

**Design:**

A community-based comparative analytical cross-sectional study.

**Participants:**

A sample of 847 randomly selected rural households.

**Results:**

The proportion of households that consumed FV at least 3 d in a week was 45 %. Members in households who owned a home garden were 1·5 times more likely to consume FV at least 3 d in a week (adjusted OR (AOR) = 1·46 (95 % CI 1·06–2·0)), compared with their counterparts who had no home gardens. Furthermore, households in which mothers had a positive attitude towards FV consumption were 1·6 times more likely to consume FV (AOR = 1·63 (95 % CI 1·17–2·27)) compared with mothers who were less positive.

**Conclusions:**

Our results suggest that food and nutrition policy measures that promote home gardens can improve consumption of diversified diets including FV among vulnerable rural households in Northern Ghana. Additionally, households with lower income may benefit from nutrition behaviour change communication campaigns directed towards increasing a positive attitude to FV intake.

Home gardening is an agricultural activity which can contribute significantly to nutrition security and livelihood of poor rural households in developing countries^(1–3)^, but it has received little research attention over the years^(1,3,4)^. The available evidence on the effect of home gardening interventions on nutritional outcomes such as vegetable consumption remains mixed because whereas some studies have reported a positive association^(5–9)^, others have reported a neutral or no effect at all^(10–13)^. The question remains as to whether households that put up such gardens truly benefit from them nutritionally. It is therefore important to further assess the effect of home gardening in different geographical settings and population groups.

The diets of many people in low- and middle-income countries (LMIC) are deficient in essential micronutrients^(14)^, emanating mainly from low consumption of nutrient-dense foods such as fruits, vegetables and animal protein^(15,16)^. Fruits and vegetables (FV) are important sources of vital nutrients that are required to prevent diet-related diseases, including, diabetes, cancer, CHD, stroke and cataract formation^(17–19)^. The WHO recommends a daily intake of at least 400 g (or five servings) of FV per person to curb non-communicable diseases^(20)^. Therefore, a sustainable means of producing and consuming FV is a necessary step towards the prevention of these diseases. It is in this light that home gardens hold a central place in the search for ways to improve household food and nutrition security. A home garden is defined as a ‘traditional land use system around a homestead, where several species of plants are grown and maintained by the household members and their products are primarily intended for the family consumption’^(21)^. They are usually sited near to the house and source of water and are managed by family members^(22)^.

A home garden can supply a family with substantial quantities of a variety of foods all year round and a source of family income, thereby providing access to diversified diets and increased purchasing power from income from sales of garden products^(23)^. Home gardening, therefore, has economic and nutritional merit but which most households are not taking advantage of due to some constraints including lack of land space and water availability.

Home gardening has the potential to increase the availability and economic access to nutrient-dense FV. However, there is paucity of evidence on the relationship between ownership of home gardens and the consumption of FV in deprived rural communities. An earlier review showed that ten out of fifteen home garden interventions in developing countries increased household production and consumption of nutrient-dense foods^(24)^. However, some randomised controlled trials on home garden interventions in some countries including Kenya, Tanzania and Uganda found no significant effects on diets^(11)^. To formulate interventions that can increase the consumption of FV, the factors that hinder their consumption need to be identified and addressed adequately. It is against this backdrop this study assessed the extent to which access to home gardens associate with the frequency of FV consumption in rural households of Northern Ghana.

## Methods

### Study setting

The study was undertaken in fifty communities in Northern Ghana where food insecurity and malnutrition including micronutrient deficiencies are prevalent. Agriculture is the main occupation for the majority of the people, while others engage in trading activities^(25)^. The main staple foods are maize, sorghum, millet and yam. There is a long dry season when it becomes very difficult for most households to access leafy green vegetables.

### Study design, population and sampling

A community-based comparative analytical cross-sectional study was conducted in rural households with children under 5 years of age. The proportion of households that consumed FV was unknown and was thus assumed to be 50 % as per common practice^(26)^. The minimum sample size was calculated with 5 % margin of error at 95 % confidence level and a design effect of 2·0. Based on these assumptions, a one-point sample size of 768 was determined. The provision of 10·0 % was made to take care of unforeseen circumstances including incomplete and damaged questionnaires. The sample size was thus adjusted to 847 households.

The eligible study households were selected from a multistage approach. First, five districts were selected from simple random sampling. In each of the selected districts, ten communities were selected using a cluster sampling procedure. A systematic random sampling technique was used to select eligible households in each community. With the help of community health volunteers, households in each community were serially numbered to obtain the total number. The number of households was then divided by the cluster sample size of 17 to give the sampling interval. The first household was selected by randomly picking any number within the sampling interval (i.e. from 1 to 17). Subsequent households were selected by adding the sampling interval to the previously selected serial number. In each household, one couple was randomly selected for an interview.

### Data collection methods

The survey was undertaken in February 2021 during the lean dry season. Interviews were conducted in the households face to face by trained enumerators using a structured questionnaire. Data were collected on socio-demographic characteristics, fathers’ involvement in child feeding activities, frequency, and reasons for consuming FV, and knowledge of mothers regarding nutritional and health benefits of FV. The enumerators collected the data electronically using smartphones.

### Study variables

The principal dependent variable was the frequency of consuming FV. The study participants were asked to recall the number of days FV were eaten in the household within past 7 d preceding the study. The frequency of consuming FV was classified as low (less than 3 d in a week) and high (at least 3 d in a week),

The key explanatory variable was whether a household had a home garden or not. Home gardening was defined as growing FV in or around the home. The respondents were also asked to provide information on the kind of crops grown in their home gardens.

The mothers’ attitudes towards FV consumption were assessed by their responses to twelve positive statements about the nutritional and health benefits of FV. A three-point Likert scale, with three response options (agree, neutral and disagree), was used. A score of −1 was given to responses that disagree, while a score of 1 was assigned for agreeing to positive attitude. Neutral (do not know) responses were scored 0. A total correct score for each respondent was then calculated and classified as positive if was at least the median score, otherwise it was considered as negative.

### Assessment of socio-economic status

The wealth index was used as a proxy for household socio-economic status which was quantified using the principal component analysis. The wealth index was based on ownership of durable economic assets, including bicycle, television, radio, motorcycle, sewing machine, telephone, cars, refrigerator, mattress, bed, computer and mobile phone^(27–29)^. The households were then categorised into one of five wealth quintiles (ranging from poorest to richest households).

### Data analysis

The data were analysed using the SPSS package (SPSS version 22.0, IBM Corp.). To obtain correct point estimates which took the cluster sampling into account, the complex samples module of SPSS was used in the analysis. In bivariate analysis, the *χ* ^2^ was used to assess associations of categorical variables, while multivariable logistic regression model was used to measure associations between independent variables and the main outcome measure. Adjusted OR (AOR) with 95 % CI were presented as measures of association. Potential confounders that were controlled for were household size, exposure to nutrition education, type of employment, educational level of mothers and fathers, household wealth index, region of residence, mothers’ attitudes towards FV consumption, and father’s involvement in childcare and feeding.

## Results

### Socio-demographic characteristics of study participants

Table 1 shows the socio-demographic characteristics of the study participants. The mean age for mothers was 29·4 ± 6·2 years. Most mothers (55·5 %) had no formal education and 26·1 % of households were within the fifth quintile of household wealth index. Respondents were mostly of the Dagomba tribe and 53·1 % of them were Muslims. Most of the households (72·1 %) had one child who was under 2 years.

**Table 1:** Socio-demographic characteristics of sample (*n* 847)

Characteristics	Frequency (*n*)	Percentage (%)
Mother’s age (years)		
Under 30 years	439	51·8
30–39	369	43·6
40–49	39	4·6
Educational level of mothers		
None	470	55·5
Low (primary and JHS)	271	32·0
High (at least SHS)	106	12·5
Ethnicity		
Dagomba	343	40·5
Kasena	117	13·8
Wala	110	13·0
Dagaaba	227	26·8
Frafra/Builsa/Kusasi	17	2·0
Nankam	33	3·9
Religion		
Christianity	397	46·9
Islam	450	53·1
Classification of household wealth index		
First quintile	107	12·6
Second quintile	159	18·8
Third quintile	171	20·2
Fourth quintile	189	22·3
Fifth quintile	221	26·1
Children under 2 years in household		
0–1	611	72·1
2–3	221	26·1
More than 3	15	1·8

### Attitudes of mothers towards the consumption of fruits and vegetables

Respondents (at least 80 %) agreed with most of the positive statements about nutritional and health benefits of FV. However, only a modest proportion of respondents disagreed with the belief that FV are good for the poor while meat and fish are for the rich (41·6 %). As many as 55·6 % of the respondents held the false belief that FV are meant for the poor (Table 2).

**Table 2:** Attitudes of respondents on the nutritional and health benefits of fruits and vegetables

	How much do you agree with the message?					
Variable	Agree		Do not know (neutral)		Disagree	
	*n*	%	*n*	%	*n*	%
Consumption of fruits and vegetables daily is important for the survival of human beings	817	96·5	18	2·1	12	1·4
Heart diseases are prevented by eating fruits and vegetables	743	87·7	81	9·6	23	2·7
Fruits and vegetables can slow down the development of some diseases	756	89·3	69	8·1	22	2·6
Fruits and vegetables are protective foods	778	91·9	48	5·7	21	2·4
Fruits and vegetables eaten whole can prevent constipation	728	86·0	85	10·0	34	4·0
Green leafy vegetables are rich in substances that help the body to make blood for children and adults	801	94·6	33	3·9	13	1·5
Fruits and vegetables, prevent heart diseases and cancer and contribute to good health in general.	688	81·2	121	14·3	38	4·5
Overcooking can destroy nutrients in fruits and vegetable	710	83·8	74	8·7	63	7·4
Quantity of fruits and vegetables consumed is important to make one healthy and strong	784	92·6	40	4·7	23	2·7
Children should be fed with fruits and vegetables just as adults	744	87·8	39	4·6	64	7·6
Fruits and vegetables are good for the poor, while meat is for the rich	471	55·6	29	3·4	347	41·0
Consumption of green leafy vegetables can prevent night blindness due to lack of vitamin A in the body	723	85·4	97	11·5	27	3·1

### Vegetable production and consumption characteristics of home gardeners

The proportion of study participants who reported owning a home garden was 40·4 %, and most of them believed the main function of home garden was to provide daily food needs. The vegetables produced and consumed included salad greens, lettuce, eggplant, pumpkin leaves, tomatoes, beans and cucumbers. Forty-seven per cent of households consumed FV at least 3 d in the week preceding the study. High prices and non-availability of FV were cited as the main barriers to their regular consumption. Under 1 % of respondents could not mention any benefit for consuming FV (Table 3).

**Table 3:** Vegetables production and consumption characteristics of home gardeners (*N* 847)

	Frequency (*n*)	Percentage (%)
Does your household own a garden?		
No	505	59·6
Yes	342	40·4
What type of garden do you have?		
Traditional	276	32·6
Improved (container/sack)	66	7·8
No garden	505	59·6
What kind of crops are cultivated in your garden?		
Crops such as salad greens, peppers, eggplant, tomatoes, beans and cucumbers mainly for consumption	206	60·2
Crops such as salad greens, peppers, eggplant, tomatoes, beans and cucumbers mainly for sale	20	5·8
Crops such as salad greens, peppers, eggplant, tomatoes, beans and cucumbers mainly for consumption and sale	116	33·9
Frequency of consuming fruits and vegetables in the past week		
Less than 3 d in a week	448	52·9
At least 3 d in a week	399	47·1
What do you think is the main function of home garden?		
Provide daily food needs	480	56·7
Income	77	9·1
Both	290	34·2
Main source of green leafy vegetables consumed in the past week		
Own production	62	7·3
Purchases	231	27·3
Both (own production and purchases)	28	3·3
Did not consume (not applicable)	526	62·1
What is/are the benefits of consuming fruits and vegetables? (Multiple responses possible)		
Protection against diseases	508	60·0
Promote growth and development	528	62·3
Supply minerals and vitamins/gives blood	495	58·4
Cannot tell	5	0·6
Barriers to regular fruit and vegetable consumption		
High prices	376	44·4
Non-available year-round	455	53·7
Does not like taste	7	0·8
Lack of different variety	9	1·1

### Predictors of fruit and vegetable consumption in rural households

Home gardening was associated with frequent consumption of FV. Mothers who had at least secondary education compared with those with no education were more likely to consume FV at least 3 d in a week. Similarly, poorest households were less likely to consume FV. Father’s involvement in childcare and feeding was positively associated with frequent consumption of FV. There were also regional differences in the consumption of FV, with the Upper East having the highest proportion of households that consumed FV (Table 4).

**Table 4:** Factors associated with fruits and vegetables consumption (bivariate analysis)

		Frequency of consuming fruits and vegetables				
		Low (less than 3 d in a week)		High (at least 3 d in a week)		
Variable	*n*	*n*	%	*n*	%	Test statistic
Does your household own a garden?						
No	505	294	58·2	211	41·8	*χ* ^*2*^ = 14·2, *P* < 0·001
Yes	342	154	45·0	188	55·0	
Household wealth index						
First tertile	266	148	55·6	118	44·4	*χ* ^(2)^ = 14·3, *P* = 0·001
Second tertile	264	158	59·8	106	40·2	
Third tertile	317	142	44·8	175	55·2	
Mother’s attitudes towards nutritional and health benefits of fruits and vegetables						
Negative	281	165	58·7	116	41·3	*χ* ^2^ = 6·2, *P* = 0·01
Positive	511	253	49·5	258	50·5	
Region of residence						
Northern	341	195	57·2	146	42·8	*χ* ^2^ = 51. 7, *P* < 0·001
Upper East	169	48	28·4	121	71·6	
Upper West	337	205	60·8	132	39·2	
Father’s involvement in childcare and feeding						
Low (less than median score)	320	191	59·7	129	40·3	*χ* ^2^ = 9·5, *P* = 0·002
High (at least median score of 7)	527	257	48·8	270	51·2	
Mother’s educational level						
None	470	265	56·4	205	43·6	*χ* ^2^ = 8·1, *P* = 0·02
Low (primary/JHS)	271	139	51·3	132	48·7	
High (at least SHS)	106	44	41·5	62	58·5	
Classification of father’s nutrition knowledge						
Low (less than median score)	390	191	49·0	199	51·0	*χ* ^2^ = 4·5, *P* = 0·04
High (at least median score)	457	257	56·2	200	43·8	

After controlling for potential confounding factors, the ownership of home gardens remained a significant independent predictor of FV consumption (Table 5). Members in households who owned a home garden were 1·5 times more likely to consume FV at least 3 d in a week (AOR = 1·46 (95 % CI 1·06–2·0)), compared with their counterparts who had no home gardens.

**Table 5:** Predictors of fruits and vegetables (FV) consumption in rural households (multivariable logistic regression analysis)

		95 % CI		
Factors	Adjusted OR (AOR)	Lower	Upper	*P*-value
Region of residence				
Upper West region	Reference	Reference	Reference	
Northern region	1·03	0·73	1·45	0·877
Upper East region	3·46	2·23	5·36	<0·001
Participation in home gardening?				
No	Reference	Reference	Reference	
Yes	1·46	1·06	2·0	0·020
Mother’s attitude towards FV consumption				
Negative	Reference	Reference	Reference	
Positive	1·63	1·17	2·27	0·004
Wealth quintiles				
Poorest	Reference	Reference	Reference	
Second tertile (middle)	2·39	1·19	4·79	0·75
Third tertile (richest)	0·64	0·32	1·28	0·04

Compared with households in the Upper West region, households in the Upper East region were 3·5 times more likely to consume FV at least 3 d in a week (AOR = 3·46 (95 % CI 2·23–5·36)). Households in which mothers had a positive attitude towards FV consumption were 1·6 times more likely to consume FV (AOR = 1·63 (95 % CI 1·17–2·27)), compared with mothers who were less positive. Compared with children from the poorest households, children from richest wealth tertile were 2·4 times more likely to consume FV (AOR = 2·39 (95 % CI 1·19–4·79)). The set of predictors accounted for 19·0 % of the variation in FV consumption (Nagelkerke R square = 0·190).

## Discussion

The aim of this paper was to analyse whether ownership of home gardens associate with FV consumption in rural households. To the best of our knowledge, the present study is the first detailed quantitative assessment of the relationship between home gardening and FV consumption in rural households of Northern Ghana. The main finding was that ownership of home gardens was positively associated with frequent consumption of FV.

### Contribution of home gardens to the consumption of micronutrient-rich fruits and vegetables

Members in households who owned a home garden were more likely to consume FV at least 3 d in a week. The possible reason for the differences in the consumption of vegetables may be due to availability of their own produce. This finding is consistent with available evidence from systematic reviews and meta-analysis which have shown positive effect of community and home gardening on the consumption of FV^(8,30–33)^. Similarly, studies in Bangladesh and Nepal showed that a home garden intervention significantly increased household vegetable production and consumption^(34,35)^. However, some randomised controlled trials on home garden interventions in some countries including Kenya, Tanzania and Uganda found no significant effects on diets^(11)^. The investigators attributed the lack of impact to the fact that many participating households were already producing vegetables and a low participation of households in training events. From Zambia, another cluster-randomised controlled trial reported there was no effect on household or child dietary diversity^(12)^. It is unclear what accounts for the contrasts between our findings and these other findings. However, the role of other determinants of FV consumption cannot be underestimated. For example, fathers’ involvement in child feeding activities was positively associated with frequent consumption of FV in households in our study sample. This may not be the case in these other countries.

### Consumption of fruits and vegetables in rural households

Frequency of consuming FV was generally low, and 47·1 % of households consumed at least 3 d in the week prior to the study. Despite the importance of FV to human health, people in many LMIC and as reported in the present study consume far less than the required five servings^(36,37)^. In a meta-analysis of the vegetable intake of 162 countries, it was found that vegetable intake in 88 % of the investigated countries is below the WHO recommended level^(38)^. This low consumption pattern of FV has been reported in many other LMIC, including Iran, Kenya and Tanzania where it was observed that 87·5 %, 94 % and 82 %, respectively, of adults consumed less than five servings a day^(39,40)^. In many other LMIC, such as Mexico and Thailand, FV are produced mainly to export to other countries^(41)^.

The most frequently cited reasons respondents gave for consuming FV were for the promotion of growth and development and protection against diseases. Two main barriers to regular FV consumption identified in this study were high prices and non-availability. High price is a common and strong barrier to the consumption of FV, especially in low-income countries^(42)^.

Adequate consumption of FV protects against diet-related and lifestyle diseases, including, diabetes, cancer, CHD, stroke and improved immunity against non-communicable diseases^(17,18)^. The significant benefits of consuming FV buttresses the urgent need to promote and encourage their consumption through public health campaigns.

### Predictors of fruit and vegetable consumption in rural households

There was a statistically significant positive association between frequent vegetable consumption and household access to home gardens, after controlling for some confounders. This is consistent with studies including meta-analysis from different settings which have reported that home gardening interventions are positively associated with increased intake of FV in eight cases^(43,44)^. Another review of twenty-three studies in 2008 found home gardens to be positively associated with intakes of FV in fourteen cases^(45)^. However, a systematic review of twenty-three home garden interventions between 1995 and 2009 found mixed results, with unclear evidence of the influence of home gardens on diets and other health indicators including stunting^(13)^.

There was also a statistically significant positive association between frequent vegetable consumption and household wealth. Relative to a household in the highest wealth tertile (richest households), lowest tertile households (poorest) were less likely to consume FV. This finding concurs very well with recent and past studies, including systematic reviews and meta-analysis which reported that people of higher income were less likely to have a low intake of FV^(37,46,47)^. Households with higher wealth may be able to prioritise and afford FV, compared with low-income families because of price restrictions. It has also been reported that the high perishable nature of FV hinders their consumption among poor families^(48)^.

Although high educational attainment was not significant in the logistic regression, the bivariate analysis showed mothers’ educational level was a good predictor for intake of more FV. Respondents with higher education (i.e. secondary and tertiary education) were more likely to eat FV at least 3 d in a week in our study sample. The waning effect of maternal education in the logistic regression may have resulted from the colinearity with household wealth index. A number of other studies have reported of high educational attainment and FV consumption^(42,46)^. The fact that a higher FV intake was associated with higher education suggests that respondents who have attained higher educational level may be more informed in decision-making during the purchases of food.

In our study, fathers’ involvement in child feeding activities was positively associated with frequent consumption of FV in households. Traditionally, fathers are expected to provide financial and logistical resources for the family^(49–51)^. Though most interventions aim to improve child feeding practices by targeting mothers, there is an emerging evidence which suggests that when fathers are involved in nutrition education sessions in LMIC such as Kenya and Ethiopia^(52,53)^, it brings about positive nutritional outcomes. The results are also in line with an earlier study that has found positive associations between fathers’ interaction with their children and vegetable consumption^(54)^.

### Study limitations

The findings in this study should be considered in the context of its limitations. Firstly, the study was cross-sectional which has the inherent inability to establish causal relationships. In view of this limitation, future research should assess the benefits of home gardening interventions using more rigorous study design. Second, the study used the 1-week dietary recall methodology which relies on respondents’ ability to remember what foods were consumed. This self-reported data may lead to measurement bias of the food consumed. The ability to recall foods may vary between individuals based on participants’ memory and concentration levels. Despite these limitations, the findings in this study add to existing evidence that home gardens can contribute to increased consumption of FV in rural communities of LMIC.

### Conclusions

FV consumption was quite low, although ownership of home gardens was positively associated with frequent consumption of these foods. Our results also suggest that food and nutrition policy measures that promote home gardens can improve adequate consumption of diversified diets among vulnerable rural households in Northern Ghana. Additionally, households with lower income may benefit from nutrition behaviour change communication campaigns directed towards increasing positive attitudes to FV intake.
